# Population-Specific Pharmacogenetic Variants Associated with Chemotherapy Response in the Bulgarian Population

**DOI:** 10.3390/ph19101504

**Published:** 2026-09-22

**Authors:** Nelly Miteva-Marcheva, Hristo Ivanov, Iglika Sotkova-Ivanova, Nikola Boyanov, Vili Stoyanova

**Affiliations:** 1 Department of Medical Genetics, Faculty of Medicine, Medical University of Plovdiv, 4000 Plovdiv, Bulgaria; 2 Center of Competence—PERIMED 2, 4002 Plovdiv, Bulgaria; 3 Medical Simulation Training Center, Medical University of Plovdiv, 4000 Plovdiv, Bulgaria; 4 Department of Gastroenterology, Pulmed University Hospital, 4000 Plovdiv, Bulgaria; 5 Sofiamed University Hospital, 1797 Sofia, Bulgaria

**Keywords:** allele frequency, Bulgarian population, germline variants, personalized medicine

## Abstract

**Background/Objectives:** Pharmacogenetic variability influences the efficacy and toxicity of anticancer therapies. However, Eastern European populations remain underrepresented in pharmacogenetic databases. This study aimed to characterize pharmacogenetic variants relevant to chemotherapy response in the Bulgarian population and compare their allele frequencies with reference populations. **Methods:** Ninety individuals were included, comprising 50 patients with colorectal, non-small cell lung, or breast cancer and 40 healthy controls. Genomic DNA and circulating cell-free DNA were analyzed by next-generation sequencing targeting pharmacogenetic genes involved in drug metabolism, transport, and DNA repair. Allele frequencies were compared with gnomAD v2.1.1 reference data. Sensitivity analyses compared Bulgarian controls with Bulgarian non-cancer and combined Southern European, Northwestern European, and Other non-Finnish European non-cancer populations. **Results:** Twenty-three pharmacogenetic variants were evaluated. Six variants remained statistically significant after Benjamini–Hochberg correction in both Fisher’s exact test and the two-proportion Z-test. Five variants—*DPYD* c.2194G>A, *XPC* c.2815C>A, *EGFR* c.1562G>A, *XRCC1* c.1196A>G, and *ERCC2* c.2251A>C—showed higher allele frequencies, whereas *ERCC2* c.934G>A was less frequent in the Bulgarian cohort. *DPYD* c.85T>C remained significant after correction in the Z-test but not in Fisher’s exact test. Sensitivity analyses confirmed the significance of *XPC* c.2815C>A in both reference-population comparisons, while *DPYD* c.85T>C remained significant only against the combined European reference population. **Conclusions:** Several pharmacogenetic variants showed distinct allele frequencies in the Bulgarian cohort. *XPC* c.2815C>A demonstrated the most robust population-specific pattern across sensitivity analyses. These findings highlight the value of locally derived pharmacogenetic data for precision oncology and pharmacogenetically guided treatment strategies.

## 1. Introduction

Pharmacogenetic differences contribute substantially to the variability in individual responses to chemotherapy, affecting both treatment effectiveness and the occurrence of adverse drug reactions. Germline genetic variation in pathways related to drug biotransformation, transport, and DNA damage response can modify systemic drug exposure, tumor sensitivity, and toxicity patterns across multiple classes of anticancer agents [1,2].

A growing body of evidence supports the clinical relevance of pharmacogenetic markers for widely used chemotherapeutic regimens, including platinum compounds, fluoropyrimidines, taxanes, anthracyclines, alkylating agents, and targeted therapies such as epidermal growth factor receptor (EGFR) inhibitors. Polymorphisms in genes encoding drug-metabolizing enzymes and transporters (e.g., *DPYD*, *MTHFR*, *ABCB1*, *ABCC2*, *ABCG2*, *SLC22A2*, *SLCO1B3*), as well as genes involved in oxidative stress regulation and DNA repair processes (e.g., *SOD2*, *XRCC1*, *ERCC2*, *XPC*, *ATM*, *TP53*), have been associated with variability in treatment response and clinical outcomes in cancer patients [3,4].

Although pharmacogenetic testing is increasingly implemented in personalized oncology, its broader clinical application is constrained by the limited availability of population-specific reference data. Publicly accessible databases, such as the Genome Aggregation Database (gnomAD), offer extensive allele frequency information but are largely based on individuals of European ancestry. Consequently, genetic profiles characteristic of Eastern European populations, including Bulgarians, are underrepresented, which may limit the applicability of pharmacogenetic data from global datasets to Eastern European populations [5].

The targeted gene panel used in this study was the Novogene Precision Medicine 2.0 (NovoPM 2.0) panel, developed and provided by Novogene Corporation Inc. Although multiple forms of genetic variation were detected, the variants considered relevant to chemotherapy metabolism in this study were limited to single nucleotide polymorphisms (SNPs). The gene panel was designed to capture pharmacogenetic markers relevant to treatment regimens commonly administered for colorectal cancer, breast cancer, and non-small cell lung cancer, which are among the most prevalent malignancies in Bulgaria and worldwide. These regimens included platinum-based agents, pyrimidine analogues, folinic acid, EGFR inhibitors, taxanes, anthracyclines, and cyclophosphamide [3].

The objective of this study was to characterize the distribution of germline pharmacogenetic variants relevant to these regimens in Bulgarian cancer patients and to assess their allele frequencies in comparison with global reference populations. Identifying population-specific patterns of pharmacogenetic variation may enhance the clinical relevance of genetic testing and support the development of more precise, locally informed approaches to pharmacogenetically guided treatment.

## 2. Results

Of the 26 patients initially enrolled with colorectal cancer, three were excluded from further analysis because they received radiotherapy without chemotherapy. Consequently, 23 patients with colorectal cancer (CRC) were included in the final evaluation. These individuals received combinations of platinum-based agents (oxaliplatin), folinic acid (leucovorin), pyrimidine analogues (5-fluorouracil, capecitabine), topoisomerase I inhibitors (irinotecan), EGFR inhibitors (panitumumab, cetuximab), and monoclonal antibodies (bevacizumab, ramucirumab) (Supplementary Table S1).

The non-small cell lung cancer (NSCLC) cohort consisted of 13 patients treated with taxanes (docetaxel, paclitaxel), platinum compounds (carboplatin, cisplatin), topoisomerase II inhibitors (etoposide), nucleoside analogues (gemcitabine), monoclonal antibodies (pembrolizumab, atezolizumab, bevacizumab, nivolumab, and ramucirumab), EGFR inhibitors (erlotinib), and tyrosine kinase inhibitors (alectinib) (Supplementary Table S2). Documented ADRs and corresponding toxicity grades are presented in Supplementary Table S2.

The third cancer-specific subgroup included 11 women with breast cancer. Treatment regimens comprised taxanes, anthracyclines (epirubicin), pyrimidine analogues (5-fluorouracil), platinum-based chemotherapeutics (carboplatin), alkylating agents (cyclophosphamide), and hormone therapy (Supplementary Table S3). ADRs observed in this group are detailed in Supplementary Table S3.

Given the overlap in chemotherapeutic agents administered across malignancies, patients who received chemotherapy (*n* = 47) were regrouped according to treatment type in order to evaluate the clinical relevance of pharmacogenetic variants. The resulting groups included patients treated with platinum-based agents (*n* = 35), pyrimidine analogues (*n* = 26), folinic acid (*n* = 21), EGFR inhibitors (*n* = 11), taxanes (*n* = 15), anthracyclines (*n* = 8), and cyclophosphamide (*n* = 8) (Supplementary Tables S4–S10). These tables summarize the genes associated with the metabolism of each drug class and the zygosity status (homozygous or heterozygous) of the detected variants.

Allele frequencies of germline pharmacogenetic variants were initially examined across the cancer subgroups and in the Bulgarian control group. However, because of the limited size of individual groups, the primary cohort-level analysis was based on the combined Bulgarian cohort (*n* = 90), with allele frequencies compared with those reported in gnomAD.

Among the 35 patients treated with platinum-based chemotherapeutics, high frequencies were observed for several pharmacogenetic variants relevant to drug metabolism, including *ABCC2* c.1249G>A (95%), *GSTP1* c.313A>G (94%), *SLCO1B3* c.699G>A (94%), and *XPC* c.1496C>T (91%). Significant differences from the Bulgarian control group were identified for *XPC* c.2815C>A, *XPC* c.1496C>T, and *SLC22A2* c.808T>G. Although the *ERCC2* c.934G>A variant did not differ significantly from the local control group, its allele frequency was significantly lower than that reported for non-cancer individuals in gnomAD (*p* = 0.0014; 0.0008) (Supplementary Table S11).

In patients receiving pyrimidine analogues (5-fluorouracil/capecitabine; *n* = 26), the allele frequency of DPYD c.2194G>A was significantly higher compared with both the healthy Bulgarian cohort and the gnomAD reference population (*p* < 0.05). *XRCC1* c.1196A>G also showed a significantly higher allele frequency compared with local controls (*p* = 0.0023) and gnomAD (*p* < 0.00001). No significant differences were detected for the remaining analyzed variants, including *MTHFR*, *DPYD*, *CYP1B1*, ABC transporters, *GSTP1*, *TP53*, and *ERCC2* polymorphisms (Supplementary Table S12).

Analysis of the subgroup treated with folinic acid (*n* = 21) focused on *MTHFR* c.1286A>C, *MTHFR* c.665C>T, and *ERCC2* c.2251A>C. Allele frequencies of these variants did not differ significantly from either the healthy control group or the gnomAD data (Supplementary Table S13).

Among patients treated with EGFR inhibitors (*n* = 11), only the *EGFR* c.1562G>A variant was detected, with an allele frequency of 0.9091. While no significant difference was observed in comparison with the local control group, the allele frequency was significantly higher than that in the gnomAD reference population (*p* ≈ 0.036) (Supplementary Table S14).

For patients receiving taxane-based therapy (*n* = 15), variants in *SOD2*, *ABCB1*, *SLCO1B3*, and *ERCC2* were evaluated. *SOD2* c.47T>C and *ABCB1* c.2677T>G were present in 67% of patients but showed no statistically significant differences from the control populations. Both *SLCO1B3* variants were detected in all individuals. However, their allele frequencies were comparable to those reported in local and global reference datasets. No significant differences were observed for *ERCC2* c.2251A>C (Supplementary Table S15).

Patients treated with anthracyclines (epirubicin) and cyclophosphamide (*n* = 8) represented the same subgroup and were therefore analyzed simultaneously. Detected variants included *CYP1B1* c.1294G>C, *ABCG2* c.421C>A, *ABCC2* c.1249G>A, *GSTP1* c.313A>G, *ATM* c.5557G>A, *TP53* c.215C>G, and *XRCC1* c.1196A>G. All patients carried *ABCC2* c.1249G>A and *GSTP1* c.313A>G, while *CYP1B1* c.1294G>C and *TP53* c.215C>G were observed in 87.5% of patients. No statistically significant differences were identified compared with the control cohorts, except for a nominally significant difference in XRCC1 c.1196A>G compared with gnomAD (Z-score *p* = 0.0366) (Supplementary Tables S16 and S17).

Finally, comparison of allele frequencies of 23 pharmacogenetic variants between the combined Bulgarian cohort (patients and controls; *n* = 90) and gnomAD revealed statistically significant differences for 10 variants (*p* < 0.05) (Table 1). Lower allele frequencies were observed for *MTHFR* c.1286A>C, *DPYD* c.85T>C, *ABCG2* c.421C>A, and *ERCC2* c.934G>A, whereas higher allele frequencies were observed for *MTHFR* c.665C>T, *DPYD* c.2194G>A, *XPC* c.2815C>A, *EGFR* c.1562G>A, *XRCC1* c.1196A>G, and *ERCC2* c.2251A>C.

Benjamini–Hochberg (BH) correction was applied separately to the 23 *p*-values from Fisher’s exact test and Z-score analysis, using a false discovery rate (FDR) threshold of 0.05. After correction, six variants remained statistically significant by both methods, with *DPYD* c.2194G>A, *XPC* c.2815C>A, *EGFR* c.1562G>A, *XRCC1* c.1196A>G, and *ERCC2* c.2251A>C showing higher allele frequencies, whereas *ERCC2* c.934G>A showed lower allele frequencies in the Bulgarian cohort compared with the gnomAD reference population. Additionally, *DPYD* c.85T>C remained significant only in the Z-score analysis (BH *q* = 0.02977), but not in Fisher’s exact test (BH *q* = 0.05356). The remaining variants with nominally significant *p*-values, including *MTHFR* c.1286A>C, *MTHFR* c.665C>T, and *ABCG2* c.421C>A, did not remain significant after BH correction (Table 1).

To assess the robustness of the observed population differences, sensitivity analyses were performed by comparing allele frequencies in the Bulgarian control group with non-cancer populations from gnomAD v2.1.1. When the Bulgarian gnomAD non-cancer population was used as the reference, *DPYD* c.85T>C and *XPC* c.2815C>A showed nominally significant differences by both Fisher’s exact test and the two-proportion Z-test; however, only *XPC* c.2815C>A remained statistically significant after Benjamini–Hochberg correction in both analyses (Table 2). When the combined Southern European (SEU), Northwestern European (NWE), and Other non-Finnish European (ONF) non-cancer population was used as the reference, *DPYD* c.85T>C and *XPC* c.2815C>A remained statistically significant after Benjamini–Hochberg correction by both methods (Table 3). These sensitivity analyses therefore confirmed the robustness of the difference observed for *XPC* c.2815C>A, whereas the significance of several other variants identified in the primary analysis was not consistently retained across population-specific reference datasets.

## 3. Discussion

The present study characterizes germline pharmacogenetic variability in Bulgarian cancer patients and identifies multiple differences in allele frequencies compared with reference populations. The observed differences between the Bulgarian cohort, local controls, and gnomAD suggest that pharmacogenetic interpretations based solely on aggregated reference populations may not fully capture population-specific variation, particularly in Eastern European populations that remain underrepresented in global genomic databases [6,7].

An additional exploratory analysis was performed to assess whether the distribution of clinically relevant pharmacogenetic alleles differed between chemotherapy-treated cancer patients and healthy controls within the Bulgarian cohort. The investigated variants were selected based on previously reported associations with chemotherapy response and treatment-related adverse drug reactions (ADRs), according to PharmGKB and supporting literature. Therefore, ADR information was used as a rationale for the selection of pharmacogenetic markers and for interpretation of their potential clinical relevance rather than as an outcome for direct genotype–toxicity correlation analysis. These comparisons aimed to identify potential differences in pharmacogenetic profiles between affected individuals and controls rather than to evaluate cancer-associated genetic susceptibility. However, the relatively small size of individual chemotherapy subgroups may have influenced allele frequency estimates and statistical comparisons. Therefore, the observed differences should be interpreted cautiously and require validation in larger independent Bulgarian cohorts.

The Benjamini–Hochberg procedure was applied to account for multiple comparisons in the main analysis. Following BH correction, six variants—*DPYD* c.2194G>A, *XPC* c.2815C>A, *EGFR* c.1562G>A, *XRCC1* c.1196A>G, *ERCC2* c.2251A>C, and *ERCC2* c.934G>A—remained statistically significant in both Fisher’s exact test and Z-score analysis. In addition, *DPYD* c.85T>C remained significant after correction in the Z-score analysis but not in Fisher’s exact test and should therefore be interpreted cautiously.

Variants within genes involved in folate metabolism displayed contrasting distribution patterns. The reduced frequency of *MTHFR* c.1286A>C in the Bulgarian cohort may have implications for its population-level relevance to fluoropyrimidine- or platinum-related toxicity. In contrast, the increased frequency of *MTHFR* c.665C>T may warrant further investigation regarding its potential relevance to treatment tolerance and adverse event risk, consistent with its reported impact on folate-dependent pathways relevant to chemotherapy response [6,8].

For fluoropyrimidine-associated genes, *DPYD* c.2194G>A emerged as a notable finding due to its higher allele frequency relative to both local controls and global datasets. This observation highlights its potential relevance to clinical dose individualization and toxicity management. Other DPYD variants examined in this study did not demonstrate population-specific differences in allele frequencies within the Bulgarian cohort. This finding does not exclude their established clinical relevance for predicting fluoropyrimidine-related toxicity, as several DPYD variants, including *DPYD2A (rs3918290), DPYD13* (rs55886062), c.2846A>T (rs67376798), and c.1129-5923C>G (HapB3; rs75017182), are recognized as clinically relevant markers for fluoropyrimidine toxicity risk [9,10].

Genes implicated in xenobiotic metabolism showed variable results. The distribution of *CYP1B1* c.1294G>C resembled that of reference populations, suggesting that this variant alone is unlikely to explain substantial interpopulation differences in anthracycline or cyclophosphamide toxicity [11,12]. Similarly, the relatively low allele frequency of *ABCG2* c.421C>A in the Bulgarian cohort reduces its potential utility as a population-specific marker, despite prior associations with chemotherapy-induced adverse reactions [6,13]. The frequency of *SLC22A2* c.808T>G was also broadly comparable with that reported in reference populations, although this variant has previously been linked to platinum-related hepatotoxicity [7,14].

Notable population-related trends were also observed among DNA repair genes. Increased frequencies of *XPC* c.2815C>A and c.1496C>T in patients treated with platinum-based regimens are consistent with previously described associations of these variants with hematologic and gastrointestinal toxicity. Likewise, the high allele frequency of *XRCC1* c.1196A>G across treatment groups suggests that this variant may warrant further investigation regarding its potential relevance to chemotherapy tolerance across multiple drug classes [7,15,16].

Differences in *ERCC2* variant distribution further emphasize regional specificity. The higher allele frequency of *ERCC2* c.2251A>C aligns with prior reports linking this variant to increased toxicity risk in platinum- and fluoropyrimidine-based therapies, whereas the comparatively low frequency of *ERCC2* c.934G>A may have implications for its population-level relevance in combined chemotherapy regimens in this population [17,18].

The high allele frequency of *EGFR* c.1562G>A in patients receiving EGFR-targeted therapy is another important finding. As this variant has been linked to skin and gastrointestinal side effects, it may warrant consideration in treatment planning, especially in settings where EGFR inhibitors or anti-VEGF drugs are commonly used [6].

The sensitivity analyses further refined the interpretation of these population differences by assessing the findings against a population-specific non-cancer reference group. When the Bulgarian control group was compared with the Bulgarian non-cancer population from gnomAD v2.1.1, *XPC* c.2815C>A remained statistically significant after Benjamini–Hochberg correction in both Fisher’s exact test and Z-score analysis, whereas *DPYD* c.85T>C was significant only before correction. In comparison with the combined Southern European, Northwestern European, and Other non-Finnish European non-cancer population, both *XPC* c.2815C>A and *DPYD* c.85T>C remained statistically significant after correction by both methods. These findings support the robustness of the *XPC* c.2815C>A difference across reference populations, while the significance of several variants identified in the primary analysis was not consistently retained when population-specific non-cancer reference groups were considered.

Overall, the pharmacogenetic profile observed in this cohort reflects a combination of overrepresented and underrepresented variants that may contribute to population-specific differences in pharmacogenetic profiles. The persistence of selected statistically significant differences after correction for multiple comparisons, together with the results of the sensitivity analyses, supports the presence of population-specific pharmacogenetic variation, although the consistency of these differences varied according to the reference population. These findings highlight the importance of considering appropriate population-specific reference datasets when interpreting pharmacogenetic variation, particularly for populations that remain underrepresented in large genomic databases.

Although comparison with gnomAD identified differences in the frequency of several pharmacogenetic variants, these findings should be interpreted in the context of the study’s limitations. In particular, the healthy control group was selected according to predefined inclusion and exclusion criteria, but its relatively small sample size limits its ability to fully represent the genetic diversity of the Bulgarian population. Similarly, the relatively small number of individuals in individual chemotherapy subgroups limits the precision of treatment-specific allele frequency estimates. Future studies involving larger, multicenter Bulgarian cohorts are warranted to further validate these findings and to clarify the clinical relevance of the observed population-specific pharmacogenetic differences.

## 4. Materials and Methods

Initially, 140 individuals were enrolled in the study. Of these, 90 participants (50 cancer patients and 40 healthy controls) completed the study procedures and were included in the final analysis. The remaining participants were excluded due to insufficient DNA quantity or quality for genetic analysis, lack of informed consent, incomplete clinical data, unsuccessful sequencing, failure to meet the predefined inclusion criteria, or withdrawal from the study, including patients who continued their treatment abroad and declined further participation in the study. The oncological group consisted of 26 patients with colorectal cancer, 13 with non-small cell lung cancer, and 11 with breast cancer. These cancer types were selected based on their high prevalence in Bulgaria and worldwide, the clinical relevance of chemotherapy-associated adverse drug reactions, and the overlap in therapeutic regimens used for their treatment. Observed adverse drug reactions (ADRs) and their severity are summarized in Supplementary Table S1, with toxicity classified according to the Common Terminology Criteria for Adverse Events (CTCAE).

The control group consisted of 40 unrelated healthy individuals of Bulgarian origin. As the study investigated germline pharmacogenetic variants, participants were enrolled across a broad age range. Individuals with a history of chronic diseases or malignant disorders were excluded from the control group. The healthy controls were included as a reference cohort for the assessment of pharmacogenetic variant frequencies.

The study protocol was reviewed and approved by the Institutional Review Board of the Scientific Ethics Committee at the Medical University of Plovdiv (approval number C–09-2/10.04.2020). Written informed consent was obtained from all participants or their legal representatives prior to enrollment. All procedures were performed in accordance with institutional ethical requirements and the principles of the Declaration of Helsinki.

Peripheral blood samples were collected in EDTA-containing Monovette tubes for the extraction of genomic DNA. For cell-free DNA (cfDNA) analysis, two Cell-Free DNA BCT (Streck) tubes were obtained from each patient. Genomic DNA was isolated using the QIAamp DNA Blood Mini Kit (QIAGEN, Venlo, The Netherlands), and DNA concentration was assessed with a NanoDrop™ One Microvolume UV–Vis spectrophotometer (Thermo Scientific™, Waltham, MA, USA). Cell-free DNA extraction was performed using the cfPure^®^ Cell-Free DNA Extraction Kit (BioChain Institute, Inc., Newark, CA, USA), and DNA was quantified using a DeNovix fluorimeter (DeNovix Inc., Wilmington, DE, USA).

Genomic DNA was obtained from 50 samples, including 10 cancer patients and 40 healthy individuals, while cfDNA was isolated from an additional 40 cancer patients. cfDNA was included in the initial study design as a liquid biopsy-based approach, as circulating cell-free DNA has the potential to provide information on both germline and somatic genetic alterations in oncology patients. In the present study, however, the analysis focused exclusively on germline pharmacogenetic variants related to chemotherapy response and treatment-associated toxicity, whereas somatic variants identified during sequencing were beyond the scope of the present study and were therefore not included in the analysis. Accordingly, the reported germline pharmacogenetic variants are presented collectively and were not stratified according to DNA source. All DNA samples were subjected to next-generation sequencing at Novogene Corporation Inc., where sequencing and initial bioinformatic processing were conducted. For cancer patients, cfDNA sequencing was performed using the Novogene Precision Medicine 2.0 (NovoPM 2.0) targeted panel, comprising 484 cancer-related genes (Supplementary Table S18). The composition of the panel was predefined by the manufacturer and was not customized for the present study. The genomic DNA samples were analyzed by whole-exome sequencing, and variants within genes included in the targeted panel that were relevant to pharmacogenetic assessment were considered for further analysis. Variants identified from genomic DNA and cfDNA were not analyzed comparatively.

The pharmacogenetic variants evaluated in the present study were selected from the sequencing results obtained from the study cohort. Selection was based on their reported relevance to chemotherapy response or treatment-related toxicity in the cancer types and treatment regimens represented in the cohort, as supported by PharmGKB and the available literature.

For the assessment of the pharmacogenetic profile of the Bulgarian cohort, allele frequencies were calculated using all included participants (*n* = 90), comprising both cancer patients and healthy controls. In the primary analysis, allele frequencies were compared with those reported in the Genome Aggregation Database (gnomAD v2.1.1), using the available reference dataset at the time of data extraction. Both cancer and non-cancer individuals were included in this primary reference dataset, consistent with the original population-level analysis. Variant nomenclature and corresponding reference and alternative alleles were verified to ensure consistency between the variants identified in our cohort and the gnomAD reference dataset.

Sensitivity analyses were subsequently performed to assess the robustness of the observed population differences using non-cancer reference populations. In these analyses, allele frequencies in the Bulgarian control group (*n* = 40) were compared separately with the Bulgarian non-cancer population available in gnomAD v2.1.1 and with a combined non-cancer European reference population comprising Southern European (SEU), Northwestern European (NWE), and Other non-Finnish European (ONF) populations. Only non-cancer individuals were included in the sensitivity analyses. The SEU, NWE, and ONF populations were combined to provide a broader non-Finnish European reference dataset.

Statistical analyses were performed using Fisher’s exact test and the two-proportion Z-test to compare allele frequencies between the Bulgarian study groups and the corresponding gnomAD reference populations, with *p* < 0.05 considered statistically significant. For each analysis, the Benjamini–Hochberg procedure was applied to account for multiple comparisons across the 23 pharmacogenetic variants. FDR-adjusted *p*-values (q-values) were calculated separately for Fisher’s exact test and the two-proportion Z-test, with q < 0.05 considered statistically significant. Results of the primary analysis are presented in Table 1, while the sensitivity analyses are presented in Table 2 and Table 3.

For each pharmacogenetic variant, allele frequencies were calculated for the clinically relevant allele associated with drug response or treatment-related toxicity, as defined according to PharmGKB and the supporting literature. Depending on the specific variant, this clinically relevant allele could correspond either to the reference or the alternative allele. The same clinically relevant allele was consistently used for comparison with the corresponding allele frequency reported in gnomAD across all analyses.

## 5. Conclusions

The findings of this study characterize the pharmacogenetic profile of a Bulgarian cancer cohort and demonstrate differences in the frequencies of clinically relevant germline variants compared with reference populations. Several of the observed differences remained statistically significant after correction for multiple comparisons in the main analysis, while sensitivity analyses demonstrated that the consistency of these differences varied according to the reference population used. The persistence of the *XPC* c.2815C>A difference across Bulgarian and broader non-Finnish European non-cancer reference populations supports its potential population-specific relevance, whereas other findings require further validation. These results highlight the importance of considering regional genetic variation when interpreting pharmacogenetic markers relevant to chemotherapy response and toxicity. Incorporation of locally derived pharmacogenetic data into existing national and international resources may improve the population specificity of pharmacogenomic models and contribute to more individualized cancer treatment strategies. Further studies involving larger, multicenter Bulgarian cohorts are warranted to validate these findings and evaluate their potential implications for the implementation of pharmacogenetic testing in oncology practice.

## Figures and Tables

**Table 1:** Allele frequencies of pharmacogenetic variants in the Bulgarian cohort and gnomAD, with statistical comparisons before and after Benjamini–Hochberg correction.

Gene	Variant	dbSNP rsID	Bulgarian AF	gnomAD AF	Fisher’s *p*-Value	Fisher’s BH q-Value	Z-Score *p*-Value	Z-Score BH q-Value
*MTHFR*	c.1286A>C	rs1801131	0.22	0.2890	**0.0485**	0.11155	**0.0477**	0.09974
*MTHFR*	c.665T>C	rs1801133	0.39	0.3149	**0.0367**	0.09379	**0.03**	0.07667
*DPYD*	c.2194G>A	rs1801160	0.12	0.04687	**0.0001**	**0.000575**	**<0.00001**	**<0.0000575**
*DPYD*	c.1627A>G	rs1801159	0.18	0.1977	0.5743	0.77699	0.5485	0.70086
*DPYD*	c.496A>G	rs2297595	0.1	0.08532	0.5029	0.72292	0.48392	0.69564
*DPYD*	c.85T>C	rs1801265	0.69	0.7718	**0.0163**	0.05356	**0.00906**	**0.02977**
*CYP1B1*	c.1294G>C	rs1056836	0.64	0.6317	0.7576	0.83830	0.8181	0.85529
*XPC*	c.2815C>A	rs2228001	0.57	0.3665	**<0.00001**	**<0.0000767**	**<0.00001**	**<0.0000575**
*XPC*	c.1496C>T	rs2228000	0.82	0.7565	0.0676	0.14135	**0.0477**	0.09974
*ABCG2*	c.421C>A	rs2231142	0.07	0.1247	**0.0315**	0.09056	**0.02642**	0.07596
*SLC22A2*	c.808T>G	rs316019	0.09	0.1015	0.7104	0.83830	0.61006	0.73849
*SOD2*	c.47T>C	rs4880	0.55	0.5161	0.3716	0.56979	0.36282	0.55632
*EGFR*	c.1562G>A	rs2227983	0.82	0.7079	**0.001**	**0.00460**	**0.00094**	**0.004324**
*ABCB1*	c.2677T>G	rs2032582	0.53	0.5389	0.7654	0.83830	0.81034	0.85529
*ABCC2*	c.1249G>A	rs2273697	0.79	0.809	0.6351	0.81152	0.5157	0.69771
*GSTP1*	c.313A>G	rs1695	0.71	0.6614	0.1803	0.29621	0.16758	0.27531
*ATM*	c.5557G>A	rs1801516	0.15	0.1136	0.1265	0.22805	0.12356	0.21861
*SLCO1B3*	c.334T>G	rs4149117	0.82	0.8082	0.8499	0.88853	0.68916	0.79253
*SLCO1B3*	c.699G>A	rs7311358	0.86	0.8083	0.1289	0.22805	0.0784	0.15027
*TP53*	c.215C>G	rs1042522	0.67	0.6682	1	1.00000	0.96012	0.96012
*XRCC1*	c.1196A>G	rs25487	0.53	0.3194	**<0.00001**	**<0.0000767**	**<0.00001**	**<0.0000575**
*ERCC2*	c.2251A>C	rs13181	0.43	0.3247	**0.0024**	**0.00920**	**0.00252**	**0.00966**
*ERCC2*	c.934G>A	rs1799793	0.54	0.71	**<0.00001**	**<0.0000767**	**<0.00001**	**<0.0000575**

Note: AF, allele frequency; BH, Benjamini–Hochberg; gnomAD, Genome Aggregation Database. Bold *p*-values indicate nominal statistical significance (*p* < 0.05), whereas bold BH q-values indicate statistical significance after correction for multiple comparisons (q < 0.05). The same clinically relevant allele was used for comparisons between the Bulgarian cohort and gnomAD.

**Table 2:** Comparison of pharmacogenetic variant allele frequencies between Bulgarian controls and the Bulgarian non-cancer population from gnomAD v2.1.1.

Gene	Variant	dbSNP rsID	Bulgarian Controls AF	Bulgarian gnomAD Non-Cancer AF	Fisher’s Exact *p*-Value	Fisher’s BH q-Value	Z-Test *p*-Value	Z BH q-Value
*MTHFR*	c.1286A>C	rs1801131	0.2375	0.3307	0.090276	0.519088	0.080444	0.462551
*MTHFR*	c.665C>T	rs1801133	0.4250	0.3525	0.192484	0.627869	0.182064	0.495710
*DPYD*	c.2194G>A	rs1801160	0.0750	0.0602	0.630233	0.902175	0.585688	0.792401
*DPYD*	c.1627A>G	rs1801159	0.1875	0.2013	0.887383	0.902175	0.762184	0.894755
*DPYD*	c.496A>G	rs2297595	0.1250	0.1193	0.860771	0.902175	0.876038	0.894755
*DPYD*	c.85T>C	rs1801265	0.6375	0.7682	**0.010400**	0.119604	**0.006692**	0.076956
*CYP1B1*	c.1294G>C	rs1056836	0.6750	0.6063	0.245122	0.627869	0.215526	0.495710
*XPC*	c.2815C>A	rs2228001	0.6625	0.4750	**0.001318**	**0.030318**	**0.000950**	**0.021856**
*XPC*	c.1496C>T	rs2228000	0.8500	0.7897	0.210662	0.627869	0.191032	0.495710
*ABCG2*	c.421C>A	rs2231142	0.0500	0.0856	0.410011	0.725404	0.259952	0.534388
*SLC22A2*	c.808T>G	rs316019	0.0375	0.0759	0.279015	0.627869	0.189945	0.495710
*SOD2*	c.47T>C	rs4880	0.5250	0.5054	0.734923	0.902175	0.729858	0.894755
*EGFR*	c.1562G>A	rs2227983	0.7750	0.7102	0.258534	0.627869	0.207222	0.495710
*ABCB1*	c.2677T>G	rs2032582	0.4625	0.5452	0.171086	0.627869	0.143958	0.495710
*ABCC2*	c.1249G>A	rs2273697	0.7750	0.7837	0.890298	0.902175	0.852848	0.894755
*GSTP1*	c.313A>G	rs1695	0.6875	0.6944	0.902175	0.902175	0.894755	0.894755
*ATM*	c.5557G>A	rs1801516	0.0625	0.1335	0.064785	0.496683	0.064100	0.462551
*SLCO1B3*	c.334T>G	rs4149117	0.8875	0.8527	0.519114	0.852830	0.386175	0.634430
*SLCO1B3*	c.699G>A	rs7311358	0.8750	0.8526	0.747652	0.902175	0.577431	0.792401
*TP53*	c.215C>G	rs1042522	0.6875	0.7415	0.300285	0.627869	0.278811	0.534388
*XRCC1*	c.1196A>G	rs25487	0.3875	0.3406	0.402877	0.725404	0.384424	0.634430
*ERCC2*	c.2251A>C	rs13181	0.4250	0.3941	0.642427	0.902175	0.578201	0.792401
*ERCC2*	c.934G>A	rs1799793	0.5625	0.5781	0.811056	0.902175	0.789736	0.894755

Note: AF, allele frequency; gnomAD, Genome Aggregation Database; BH, Benjamini–Hochberg. gnomAD data were restricted to non-cancer individuals from gnomAD v2.1.1. Fisher’s exact test and the two-proportion Z-test were two-sided. Benjamini–Hochberg correction was applied separately to the 23 Fisher’s exact test *p*-values and the 23 Z-test *p*-values. Bold values indicate *p*-values or BH-adjusted q-values < 0.05.

**Table 3:** Comparison of pharmacogenetic variant allele frequencies between Bulgarian controls and the combined SEU + NWE + ONF non-cancer population from gnomAD v2.1.1.

Gene	Variant	dbSNP rsID	Bulgarian Controls AF	Combined SEU + NWE + ONF Non-Cancer AF	Fisher’s *p*-Value	Fisher’s BH q-Value	Z-Test *p*-Value	Z BH q-Value
*MTHFR*	c.1286A>C	rs1801131	0.2375	0.3082	0.184335	0.414384	0.171021	0.378322
*MTHFR*	c.665C>T	rs1801133	0.4250	0.3535	0.198184	0.414384	0.180936	0.378322
*DPYD*	c.2194G>A	rs1801160	0.0750	0.0496	0.294207	0.544069	0.296084	0.523841
*DPYD*	c.1627A>G	rs1801159	0.1875	0.1990	0.889077	0.889077	0.796929	0.796929
*DPYD*	c.496A>G	rs2297595	0.1250	0.0962	0.344699	0.545124	0.383079	0.587388
*DPYD*	c.85T>C	rs1801265	0.6375	0.7843	**0.002545**	**0.029268**	**0.001426**	**0.016399**
*CYP1B1*	c.1294G>C	rs1056836	0.6750	0.5698	0.070043	0.268497	0.057439	0.220184
*XPC*	c.2815C>A	rs2228001	0.6625	0.4130	**0.000011**	**0.000253**	**0.000006**	**0.000138**
*XPC*	c.1496C>T	rs2228000	0.8500	0.7538	0.050679	0.268497	**0.045950**	0.220184
*ABCG2*	c.421C>A	rs2231142	0.0500	0.1059	0.142141	0.366797	0.104211	0.299607
*SLC22A2*	c.808T>G	rs316019	0.0375	0.1019	0.062163	0.268497	0.056860	0.220184
*SOD2*	c.47T>C	rs4880	0.5250	0.5020	0.737598	0.807845	0.680892	0.730136
*EGFR*	c.1562G>A	rs2227983	0.7750	0.7416	0.609157	0.775975	0.494991	0.669694
*ABCB1*	c.2677T>G	rs2032582	0.4625	0.5490	0.143529	0.366797	0.119974	0.306599
*ABCC2*	c.1249G>A	rs2273697	0.7750	0.7983	0.578631	0.775975	0.604492	0.722247
*GSTP1*	c.313A>G	rs1695	0.6875	0.6619	0.723035	0.807845	0.628041	0.722247
*ATM*	c.5557G>A	rs1801516	0.0625	0.1427	**0.037270**	0.268498	**0.040325**	0.220183
*SLCO1B3*	c.334T>G	rs4149117	0.8875	0.8597	0.627905	0.775975	0.474529	0.669694
*SLCO1B3*	c.699G>A	rs7311358	0.8750	0.8600	0.871694	0.889077	0.698391	0.730136
*TP53*	c.215C>G	rs1042522	0.6875	0.7398	0.307517	0.544068	0.286271	0.528757
*XRCC1*	c.1196A>G	rs25487	0.3875	0.3584	0.641023	0.775975	0.587790	0.722247
*ERCC2*	c.2251A>C	rs13181	0.4250	0.3729	0.355515	0.545124	0.335135	0.550579
*ERCC2*	c.934G>A	rs1799793	0.5625	0.6505	0.101644	0.333972	0.099034	0.299607

Note: AF, allele frequency; gnomAD, Genome Aggregation Database; SEU, Southern European; NWE, Northwestern European; ONF, Other non-Finnish European; BH, Benjamini–Hochberg. gnomAD data were restricted to non-cancer individuals from gnomAD v2.1.1. The combined European population comprised SEU, NWE, and ONF populations. Fisher’s exact test and the two-proportion Z-test were two-sided. Benjamini–Hochberg correction was applied separately to the 23 Fisher’s exact test *p*-values and the 23 Z-test *p*-values. Bold values indicate *p*-values or BH-adjusted q-values < 0.05.

## Data Availability

The data presented in this study are available on request from the corresponding author due to ethical and privacy restrictions.

## Supplementary Materials

The following supporting information can be downloaded at https://www.mdpi.com/article/10.3390/ph19101504/s1, Table S1: Clinical characteristics and chemotherapy-related adverse drug reactions (ADRs) observed in patients with colorectal cancer (CRC). Toxicity severity was graded according to the Common Terminology Criteria for Adverse Events (CTCAE), with grades ranging from 1 to 4; Table S2: Clinical characteristics and chemotherapy-related adverse drug reactions (ADRs) observed in patients with non-small cell lung cancer (NSCLC). Toxicity severity was graded according to the Common Terminology Criteria for Adverse Events (CTCAE), with grades ranging from 1 to 4; Table S3: Clinical characteristics and chemotherapy-related adverse drug reactions (ADRs) observed in patients with breast cancer. Toxicity severity was graded according to the Common Terminology Criteria for Adverse Events (CTCAE), with grades ranging from 1 to 4; Table S4: Pharmacogenetic variants in genes associated with the metabolism and response to platinum-based chemotherapeutic agents among treated patients; Table S5: Pharmacogenetic variants in genes associated with the metabolism and response to pyrimidine analogues among treated patients; Table S6: Pharmacogenetic variants in genes associated with the metabolism and response to folinic acid among treated patients; Table S7: Pharmacogenetic variants in genes associated with the metabolism and response to EGFR inhibitors among treated patients; Table S8: Pharmacogenetic variants in genes associated with the metabolism and response to taxanes among treated patients; Table S9: Pharmacogenetic variants in genes associated with the metabolism and response to Farmorubicin among treated patients; Table S10: Pharmacogenetic variants in genes associated with the metabolism and response to Cyclophosphamide among treated patients; Table S11: Genes associated with the metabolism and response to platinum-based chemotherapeutic agents; Table S12: Genes associated with the metabolism and response to 5-FU/Capecitabine; Table S13: Genes associated with the metabolism and response to Leucovorin; Table S14: Genes associated with the metabolism and response to EGFR inhibitors; Table S15: Genes associated with the metabolism and response to taxanes; Table S16: Genes associated with the metabolism and response to anthracyclines (Farmorubicin); Table S17: Genes associated with the metabolism and response to Cyclophosphamide; Table S18: Complete list of genes included in the Novogene Precision Medicine 2.0 (NovoPM 2.0) targeted sequencing panel.

## Abbreviations

The following abbreviations are used in this manuscript:

5-FU	5-Fluorouracil
ABCB	ATP-Binding Cassette Subfamily B
ABCC	ATP-Binding Cassette Subfamily C
ABCG	ATP-Binding Cassette Superfamily G
ADR	Adverse Drug Reaction
ATM	Ataxia-Telangiectasia Mutated
BH	Benjamini–Hochberg
cfDNA	Cell-Free DNA
CRC	Colorectal Carcinoma
CTCAE	Common Terminology Criteria for Adverse Events
CYP	Cytochrome P450
DNA	Deoxyribonucleic Acid
DPYD	Dihydropyrimidine Dehydrogenase Gene
EDTA	Ethylenediaminetetraacetic Acid
EGFR	Epidermal Growth Factor Receptor
ERCC	Excision Repair Cross Complementation
FDR	False Discovery Rate
GSTP	Glutathione S-Transferase pi
MTHFR	Methylenetetrahydrofolate Reductase
NSCLC	Non-Small Cell Lung Carcinoma
NEW	Northwestern European
ONF	Other non-Finnish European
SEU	Southern European
SLC	Solute Carrier Gene
SNP	Single Nucleotide Polymorphism
SOD	Superoxide Dismutase
TP53	Tumor Protein 53
XPC	Xeroderma Pigmentosum, Complementation Group C
XRCC	X-ray Repair Cross-Complementing Protein
