# Supplementary material for: Population-Specific Pharmacogenetic Variants Associated with Chemotherapy Response in the Bulgarian Population
Source: Pharmaceuticals (Basel). 2026 Sep 22;19(10):1504. doi: 10.3390/ph19101504 (PMC13708335; doi:10.3390/ph19101504)
Supplement: Supplementary file 1 [file pharmaceuticals-19-01504-s001.zip › Supplementary Table S18.pdf]

Supplementary Table S18
Complete list of genes included in the Novogene Precision Medicine 2.0 (NovoPM 2.0) targeted sequencing panel.

SNV | INDEL | CNV						
ABCB1	ABCC2	ABCC4	ABCG2	ABL1	ABL2	ABRAXAS1
ACVR1B	ADGRA2	AKT1	AKT2	AKT3	ALK	ALOX12B
AMER1	APC	APCDD1	AR	ARAF	ARFRP1	ARID1A
ARID1B	ARID2	ASXL1	ATM	ATR	ATRX	AURKA
AURKB	AXIN1	AXIN2	AXL	BACH1	BAP1	BARD1
BCL2	BCL2A1	BCL2L1	BCL2L2	BCL6	BCOR	BCORL1
BCR	BLM	BMPR1A	BRAF	BRCA1	BRCA2	BRD4
BRIP1	BTG1	BTG2	BTK	C8orf34	CALR	CARD11
CASP8	CBFB	CBL	CCND1	CCND2	CCND3	CCNE1
CD22	CD274	CD70	CD74	CD79A	CD79B	CDC73
CDH1	CDH2	CDH20	CDH5	CDK12	CDK4	CDK6
CDK8	CDKN1A	CDKN1B	CDKN2A	CDKN2B	CDKN2C	CEBPA
CFTR	CHD2	CHD4	CHEK1	CHEK2	CHUK	CIC
CRBN	CREBBP	CRKL	CRLF2	CSF1R	CSF3R	CTCF
CTNNA1	CTNNB1	CUL3	CUL4A	CUL4B	CXCR4	CYLD
CYP17A1	CYP1B1	CYP2C19	CYP2C8	CYP2D6	CYP3A4	CYP3A5
DAXX	DDR1	DDR2	DICER1	DIS3	DNMT3A	DOT1L
DPYD	EED	EGFR	EMSY	EP300	EPCAM	EPHA3
EPHA5	EPHA6	EPHA7	EPHB1	EPHB4	EPHB6	ERBB2
ERBB3	ERBB4	ERCC1	ERCC2	ERCC3	ERCC4	ERG
ERRFI1	ESR1	ESR2	ETV1	ETV4	ETV5	ETV6
EWSR1	EZH2	EZR	FAM46C	FANCA	FANCC	FANCD2
FANCE	FANCF	FANCG	FANCI	FANCL	FANCM	FAS
FAT1	FAT3	FBXW7	FCGR3A	FGF10	FGF12	FGF14
FGF19	FGF23	FGF3	FGF4	FGF6	FGF7	FGFR1
FGFR2	FGFR3	FGFR4	FH	FLCN	FLT1	FLT3
FLT4	FOXL2	FOXP1	FRS2	FUBP1	GABRA6	GALNT12
GATA1	GATA2	GATA3	GATA4	GATA6	GEN1	GID4
GLI1	GNA11	GNA13	GNAQ	GNAS	GREM1	GRIN2A
GRM3	GSK3B	GSTP1	H3F3A	HDAC1	HDAC2	HFE
HGF	HLA-A	HLA-B	HLA-C	HNF1A	HOXB13	HRAS
HSD3B1	HSP90AA1	ID3	IDH1	IDH2	IDO1	IDO2
IGF1	IGF1R	IGF2	IGF2R	IKBKE	IKZF1	IL7R
INHBA	INPP4B	INSR	IRF2	IRF4	IRS2	ITPA
JAK1	JAK2	JAK3	JUN	KAT6A	KDM5A	KDM5C
KDM6A	KDR	KEAP1	KEL	KIT	KLHL6	KMT2A
KMT2C	KMT2D	KRAS	LMO1	LRP1B	LRP2	LRP6
LTK	LYN	LZTR1	MAF	MAGI2	MAN1B1	MAP2K1
MAP2K2	MAP2K4	MAP3K1	MAP3K13	MAPK1	MAX	MC1R
MCL1	MDM2	MDM4	MED12	MEF2B	MEN1	MERTK
MET	MITF	MKNK1	MKNK2	MLH1	MLH3	MPL
MRE11	MSH2	MSH3	MSH6	MST1R	MTAP	MTHFR
MTOR	MUTYH	MYB	MYC	MYCL	MYCN	MYD88
NBN	NCOR1	NF1	NF2	NFE2L2	NFKBIA	NKX2-1

Supplementary Table S18
Complete list of genes included in the Novogene Precision Medicine 2.0 (NovoPM 2.0) targeted sequencing panel.

NOTCH1	NOTCH2	NOTCH3	NOTCH4	NPM1	NQO1	NRAS
NRP2	NSD1	NSD2	NSD3	NT5C2	NTHL1	NTRK1
NTRK2	NTRK3	NUDT1	NUP93	NUTM1	P2RY8	PAK3
PAK5	PALB2	PARP1	PARP2	PARP3	PARP4	PAX5
PBRM1	PDCD1	PDCD1LG2	PDGFRA	PDGFRB	PDK1	PHLPP2
PIK3C2B	PIK3C2G	PIK3C3	PIK3CA	PIK3CB	PIK3CG	PIK3R1
PIK3R2	PIM1	PLCG2	PMS2	PNRC1	POLD1	POLE
PPARG	PPM1D	PPP2R1A	PPP2R2A	PRDM1	PREX2	PRKAR1A
PRKCI	PRKDC	PRKN	PRSS1	PRSS8	PTCH1	PTCH2
PTEN	PTPN11	PTPRD	PTPRO	QKI	RAC1	RAD21
RAD50	RAD51	RAD51B	RAD51C	RAD51D	RAD52	RAD54L
RAF1	RANBP2	RARA	RB1	RBM10	REL	RET
RICTOR	RNF43	ROS1	RPA1	RPTOR	RSPO2	RUNX1
RUNX1T1	SDC4	SDHA	SDHAF2	SDHB	SDHC	SDHD
SETD2	SF3B1	SGK1	SH2B3	SLC19A1	SLC22A2	SLC34A2
SLCO1B3	SLIT2	SMAD2	SMAD3	SMAD4	SMARCA4	SMARCB1
SMARCD1	SMO	SNCAIP	SOCS1	SOD2	SOX10	SOX2
SOX9	SPEN	SPINK1	SPOP	SPTA1	SRC	STAG2
STAT3	STAT4	STK11	SUFU	SULT1A1	SYK	TAF1
TBX3	TDO2	TEK	TERC	TERT	TET2	TGFBR2
TIPARP	TMEM127	TMPRSS2	TNF	TNFAIP3	TNFRSF14	TNKS
TNKS2	TOP1	TOP2A	TP53	TP53BP1	TPMT	TRRAP
TSC1	TSC2	TSHR	TYMS	TYRO3	U2AF1	UGT1A1
UMPS	VEGFA	VHL	WISP3	WRN	WT1	XPC
XPO1	XRCC1	XRCC2	XRCC3	ZBTB2	ZNF217	ZNF703
ZNRF3						

Fusions

ALK	BCL2	BCR	BRAF	BRCA1	BRCA2	BRD4
CD74	EGFR	ERBB2	ETV1	ETV4	ETV5	ETV6
EWSR1	EZR	FGFR1	FGFR2	FGFR3	IDH1	IDH2
KIT	KMT2A	MET	MSH2	MYB	MYC	NOTCH2
NTRK1	NTRK2	NTRK3	NUTM1	PDGFRA	RAF1	RARA
RET	ROS1	RSPO2	SDC4	SLC34A2	TERC	TERT
TMPRSS2						
